# Supplementary material for: From sequence to enzyme mechanism using multi-label machine learning
Source: BMC Bioinformatics. 2014 May 19;15:150. doi: 10.1186/1471-2105-15-150 (PMC4229970; doi:10.1186/1471-2105-15-150)
Supplement: Additional file 2 — Java code of ml2db. Additional file ml2db_code.tar.gz contains the Java source code to run the multi-label machine learning experiments and save the results to database. The code’s Javadoc is included. [file 1471-2105-15-150-S2.zip › additional file 2/ml2db/ml2db/doc/test/ml2db/dataharness/DataOne.html]

DataOne


JavaScript is disabled on your browser.


- Overview
- Package
- Class
- Use
- Tree
- Deprecated
- Index
- Help

- Prev Class
- Next Class

- Frames
- No Frames

- All Classes

- Summary:
- Nested |
- Field |
- Constr |
- Method

- Detail:
- Field |
- Constr |
- Method


test.ml2db.dataharness

## Class DataOne

- java.lang.Object
- - test.ml2db.dataharness.DataOne

- ---

    

  ```
  public class DataOne
  extends java.lang.Object
  ```

  Class

  Version:
  :   25 Feb 2011

  Author:
  :   Luna De Ferrari ldf(at)st-andrews.ac.uk

- - ### Field Summary

    Fields

    | Modifier and Type | Field and Description |
    | --- | --- |
    | `static java.lang.String` | `arffEmptyInstance` |
    | `static java.lang.String` | `arffInstance1` |
    | `static java.lang.String` | `arffInstance1b` |
    | `static java.lang.String` | `arffInstance2` |
    | `static java.lang.String` | `arffInstance4` |
    | `static java.lang.String` | `arffInstanceAttVoid` |
    | `static java.lang.String` | `arffInstanceClassVoid` |
    | `static java.lang.String` | `ATT1` |
    | `static java.lang.String` | `ATT1_TO_STRING` |
    | `static java.lang.String` | `ATT2` |
    | `static java.lang.String` | `ATT2_TO_STRING` |
    | `static java.lang.String` | `ATT3` |
    | `static java.lang.String` | `ATT3_TO_STRING` |
    | `static java.lang.String` | `ATT4` |
    | `static java.lang.String` | `ATT4_TO_STRING` |
    | `static java.lang.String` | `ATT5` |
    | `static java.lang.String` | `ATT5_TO_STRING` |
    | `static java.lang.String` | `CA_CLASS1` |
    | `static java.lang.String` | `CA_CLASS2` |
    | `static java.lang.String` | `CA_CLASS3` |
    | `static java.lang.String` | `CA_CLASS4` |
    | `static java.lang.String` | `CLASS_TO_STRING` |
    | `static java.lang.String` | `CLASS1` |
    | `static java.lang.String` | `CLASS2` |
    | `static java.lang.String` | `CLASS3` |
    | `static java.lang.String` | `CLASS4` |
    | `static java.lang.String` | `DATASET_NAME` |
    | `static java.lang.String` | `FA_ATT1` |
    | `static java.lang.String` | `FA_ATT2` |
    | `static java.lang.String` | `FA_ATT3` |
    | `static java.lang.String` | `FA_ATT4` |
    | `static java.lang.String` | `FA_ATT5` |
    | `static java.lang.String` | `INST1` |
    | `static java.lang.String` | `INST2` |
    | `static java.lang.String` | `INST3` |
    | `static java.lang.String` | `INST4` |
    | `static java.lang.String` | `INSTATTVOID` |
    | `static java.lang.String` | `INSTCLASSATTVOID` |
    | `static java.lang.String` | `INSTCLASSVOID` |
    | `static int` | `NUMBER_OF_INSTANCES_IN_TEST_ARFF` |
  - ### Constructor Summary

    Constructors

    Constructor and Description

    `DataOne()`
  - ### Method Summary

    Methods

    | Modifier and Type | Method and Description |
    | --- | --- |
    | `static java.util.Vector<java.lang.String>` | `attributesColumn()` |
    | `static java.util.Vector<java.lang.String>` | `attributesValues()` Results of a select distinct instance, attribute query |
    | `static java.util.Vector<java.lang.String>` | `classColumn()` |
    | `static java.util.Vector<java.lang.String>` | `classValues()` Results of a select distinct instance, class query |
    | `static java.util.Vector<java.lang.String>` | `instancesColumn()` |
    | `static java.util.Vector<java.lang.String>` | `instancesValues()` Results of a select distinct instance, class query |

    - ### Methods inherited from class java.lang.Object

      `equals, getClass, hashCode, notify, notifyAll, toString, wait, wait, wait`

- - ### Field Detail


    - #### NUMBER\_OF\_INSTANCES\_IN\_TEST\_ARFF

      ```
      public static int NUMBER_OF_INSTANCES_IN_TEST_ARFF
      ```


    - #### arffInstance1

      ```
      public static final java.lang.String arffInstance1
      ```

      See Also:
      :   Constant Field Values


    - #### arffInstance1b

      ```
      public static final java.lang.String arffInstance1b
      ```

      See Also:
      :   Constant Field Values


    - #### arffInstance2

      ```
      public static final java.lang.String arffInstance2
      ```

      See Also:
      :   Constant Field Values


    - #### arffInstance4

      ```
      public static final java.lang.String arffInstance4
      ```

      See Also:
      :   Constant Field Values


    - #### arffEmptyInstance

      ```
      public static final java.lang.String arffEmptyInstance
      ```

      See Also:
      :   Constant Field Values


    - #### arffInstanceClassVoid

      ```
      public static final java.lang.String arffInstanceClassVoid
      ```

      See Also:
      :   Constant Field Values


    - #### arffInstanceAttVoid

      ```
      public static final java.lang.String arffInstanceAttVoid
      ```

      See Also:
      :   Constant Field Values


    - #### ATT1

      ```
      public static final java.lang.String ATT1
      ```

      See Also:
      :   Constant Field Values


    - #### ATT2

      ```
      public static final java.lang.String ATT2
      ```

      See Also:
      :   Constant Field Values


    - #### ATT3

      ```
      public static final java.lang.String ATT3
      ```

      See Also:
      :   Constant Field Values


    - #### ATT4

      ```
      public static final java.lang.String ATT4
      ```

      See Also:
      :   Constant Field Values


    - #### ATT5

      ```
      public static final java.lang.String ATT5
      ```

      See Also:
      :   Constant Field Values


    - #### FA\_ATT1

      ```
      public static final java.lang.String FA_ATT1
      ```

      See Also:
      :   Constant Field Values


    - #### ATT1\_TO\_STRING

      ```
      public static final java.lang.String ATT1_TO_STRING
      ```

      See Also:
      :   Constant Field Values


    - #### FA\_ATT2

      ```
      public static final java.lang.String FA_ATT2
      ```

      See Also:
      :   Constant Field Values


    - #### ATT2\_TO\_STRING

      ```
      public static final java.lang.String ATT2_TO_STRING
      ```

      See Also:
      :   Constant Field Values


    - #### FA\_ATT3

      ```
      public static final java.lang.String FA_ATT3
      ```

      See Also:
      :   Constant Field Values


    - #### ATT3\_TO\_STRING

      ```
      public static final java.lang.String ATT3_TO_STRING
      ```

      See Also:
      :   Constant Field Values


    - #### FA\_ATT4

      ```
      public static final java.lang.String FA_ATT4
      ```

      See Also:
      :   Constant Field Values


    - #### ATT4\_TO\_STRING

      ```
      public static final java.lang.String ATT4_TO_STRING
      ```

      See Also:
      :   Constant Field Values


    - #### FA\_ATT5

      ```
      public static final java.lang.String FA_ATT5
      ```

      See Also:
      :   Constant Field Values


    - #### ATT5\_TO\_STRING

      ```
      public static final java.lang.String ATT5_TO_STRING
      ```

      See Also:
      :   Constant Field Values


    - #### CLASS\_TO\_STRING

      ```
      public static final java.lang.String CLASS_TO_STRING
      ```

      See Also:
      :   Constant Field Values


    - #### CA\_CLASS1

      ```
      public static final java.lang.String CA_CLASS1
      ```

      See Also:
      :   Constant Field Values


    - #### CA\_CLASS2

      ```
      public static final java.lang.String CA_CLASS2
      ```

      See Also:
      :   Constant Field Values


    - #### CA\_CLASS3

      ```
      public static final java.lang.String CA_CLASS3
      ```

      See Also:
      :   Constant Field Values


    - #### CA\_CLASS4

      ```
      public static final java.lang.String CA_CLASS4
      ```

      See Also:
      :   Constant Field Values


    - #### CLASS1

      ```
      public static final java.lang.String CLASS1
      ```

      See Also:
      :   Constant Field Values


    - #### CLASS2

      ```
      public static final java.lang.String CLASS2
      ```

      See Also:
      :   Constant Field Values


    - #### CLASS3

      ```
      public static final java.lang.String CLASS3
      ```

      See Also:
      :   Constant Field Values


    - #### CLASS4

      ```
      public static final java.lang.String CLASS4
      ```

      See Also:
      :   Constant Field Values


    - #### INST1

      ```
      public static final java.lang.String INST1
      ```

      See Also:
      :   Constant Field Values


    - #### INST2

      ```
      public static final java.lang.String INST2
      ```

      See Also:
      :   Constant Field Values


    - #### INST3

      ```
      public static final java.lang.String INST3
      ```

      See Also:
      :   Constant Field Values


    - #### INST4

      ```
      public static final java.lang.String INST4
      ```

      See Also:
      :   Constant Field Values


    - #### INSTCLASSATTVOID

      ```
      public static final java.lang.String INSTCLASSATTVOID
      ```

      See Also:
      :   Constant Field Values


    - #### INSTCLASSVOID

      ```
      public static final java.lang.String INSTCLASSVOID
      ```

      See Also:
      :   Constant Field Values


    - #### INSTATTVOID

      ```
      public static final java.lang.String INSTATTVOID
      ```

      See Also:
      :   Constant Field Values


    - #### DATASET\_NAME

      ```
      public static final java.lang.String DATASET_NAME
      ```

      See Also:
      :   Constant Field Values
  - ### Constructor Detail


    - #### DataOne

      ```
      public DataOne()
      ```
  - ### Method Detail


    - #### attributesColumn

      ```
      public static java.util.Vector<java.lang.String> attributesColumn()
      ```


    - #### attributesValues

      ```
      public static java.util.Vector<java.lang.String> attributesValues()
      ```

      Results of a select distinct instance, attribute query

      Returns:


    - #### classColumn

      ```
      public static java.util.Vector<java.lang.String> classColumn()
      ```


    - #### classValues

      ```
      public static java.util.Vector<java.lang.String> classValues()
      ```

      Results of a select distinct instance, class query

      Returns:


    - #### instancesColumn

      ```
      public static java.util.Vector<java.lang.String> instancesColumn()
      ```


    - #### instancesValues

      ```
      public static java.util.Vector<java.lang.String> instancesValues()
      ```

      Results of a select distinct instance, class query

      Returns:


- Overview
- Package
- Class
- Use
- Tree
- Deprecated
- Index
- Help

- Prev Class
- Next Class

- Frames
- No Frames

- All Classes

- Summary:
- Nested |
- Field |
- Constr |
- Method

- Detail:
- Field |
- Constr |
- Method
